# Endoscopic Retrograde Cholangiopancreatography in Pediatric Pancreas Divisum: Systematic Review

**DOI:** 10.1055/a-2977-6424

**Published:** 2026-10-27

**Authors:** Tareq Alsaleh, Saurabh Chandan, Shahab R. Khan, Sagar Pathak, Abdul Mohammed, Abdullah Abbasi, Maham Hayat, Natalie Cosgrove, Deepanshu Jain, Kambiz Kadkhodayan, Dennis Yang, Ojasvini C. Chandan, Muhammad K. Hasan, John George, Mustafa A. Arain

**Affiliations:** 1 Internal Medicine 440172 AdventHealth OrlandoOrlandoFloridaUnited States; 2 Advanced Endoscopy 23530 Houston MethodistHoustonTexasUnited States; 3 Internal Medicine 1861 Brigham and Women’s HospitalBostonMassachusettsUnited States; 4 Advanced Endoscopy 23331 Loma Linda University HealthLoma LindaCaliforniaUnited States; 5 Center for Interventional Endoscopy 440172 AdventHealth OrlandoOrlandoFloridaUnited States; 6 Advanced EndoscopyUniversity of IowaIowa CityIowaUnited States; 7 Center for Interventional Endoscopy 558924 AdventHealth Central FloridaOrlandoFloridaUnited States; 8 Center For Interventional Endoscopy 440172 AdventHealth OrlandoOrlandoFloridaUnited States; 9 Department of Pediatric GastroenterologyTexas Children’s Hospital West CampusHoustonTexasUnited States; 10 Center for Interventional Gastroenterology at UTHealth 12339 University of Texas McGovern Medical SchoolHoustonTexasUnited States

**Keywords:** endoscopic retrograde cholangiopancreatography, pediatric endoscopy, pancreas

## Abstract

**Background** Pancreas divisum (PD) may be associated with recurrent acute pancreatitis (RAP) or chronic pancreatitis (CP), but data on ERCP outcomes in children with PD are limited. We conducted a systematic review of the clinical, technical, and safety outcomes of ERCP for PD in children.

**Materials and Methods** Multiple databases were searched through May 2025. The primary outcome was clinical response, as defined by each individual study; secondary outcomes were symptom resolution, technical success, and post-ERCP pancreatitis (PEP). Because outcome definitions and interventions varied and no study included a control group, outcomes were summarized narratively and were not pooled.

**Results** Eight retrospective studies including 247 patients were included. Clinical response ranged from 65.0 to 93.3% across six studies, symptom resolution from 19.1 to 54.1%, and technical success from 77.8 to 100%. Reported PEP ranged from 7.9 to 20.7%, with mixed patient and procedure denominators. Response ranged from 80.0 to 100% in RAP and 60.0 to 87.9% in CP, and was higher in RAP in three of four studies reporting both. Outcome definitions differed for every outcome examined.

**Conclusions** The available pediatric literature cannot establish whether ERCP is effective in children with PD. Every study was uncontrolled and retrospective, and outcome definitions were not comparable. The reported clinical response was frequent, particularly in RAP, but may in part reflect regression to the mean, and the PEP risk warrants caution. Given the low certainty of evidence and risk of adverse events, in particular PEP, further prospective studies are needed to define the role of ERCP in this population. A core set of outcome definitions and prospective controlled studies are required to determine efficacy.

## Introduction

Pancreas divisum (PD) is the most common pancreatic congenital anomaly, occurring in 6–8% of the population. While the etiologic role of PD in the development of pancreatitis is incompletely characterized, it is described as a risk factor for recurrent acute pancreatitis (RAP) and chronic pancreatitis (CP) in children. 1 2 PD occurs as a result of failure of fusion of the ductal systems of the dorsal and ventral pancreatic buds during embryogenesis, leading to drainage predominantly via the minor papilla through a dominant dorsal pancreatic duct, while a smaller ventral duct drains through the major papilla. 1 Complete (classic) PD, found in approximately 70% of cases, is characterized by a short ventral (Wirsung) duct draining into the major papilla and a longer dorsal (Santorini) duct draining into the minor papilla, with no communication between the two ducts. 3 4 Incomplete divisum, present in approximately 20% of patients, occurs when there is a small communicating branch between the accessory and main duct.

While observational data have shown that the incidence of pancreatitis is similar in patients with PD compared to patients with normal duct variants, the exact role of PD as an etiologic factor for pancreatitis remains unclear. Given the frequent presence of genetic mutations in patients with pancreatitis and PD, PD is considered to be a cofactor rather than a direct etiologic cause in the pathogenesis of RAP and CP in both adults and children. 5 6 7 A proposed mechanism for the underlying risk of pancreatitis is functional obstruction at the smaller minor papilla, resulting in dorsal duct hypertension, 8 which is supported by earlier manometry studies showing increased pressures in the dorsal duct and minor papilla in comparison to the ventral duct and major papilla in PD. 9

Pancreatic endotherapy performed using endoscopic retrograde cholangiopancreatography (ERCP) with minor papilla sphincterotomy (or papillotomy), balloon dilation, stent placement, or through a combination of these interventions, is proposed as a strategy to relieve outflow obstruction at the minor papilla in the symptomatic management of PD. 1 10 11 However, comparative evidence has been limited until recently. In the multicenter, sham-controlled, double-blind SHARP randomized clinical trial of 148 adults with otherwise unexplained acute recurrent pancreatitis and PD, ERCP with minor papillotomy did not significantly reduce RAP compared with sham ERCP (adjusted hazard ratio, 0.83 [95% CI, 0.49–1.41]), and pancreatitis within 30 days occurred more often after minor papillotomy. Notably, pediatric patients were not included. 12 Pediatric pancreatitis differs from adult disease in several clinically relevant ways: alcohol and tobacco exposure, major contributors to adult recurrent and CP, are uncommon in children, whereas genetic and obstructive risk factors predominate. 13 These genetic variants, such as CFTR, SPINK1, PRSS1, or CTRC, may result in more viscous secretions and debris and may modify the response to ductal drainage procedures. 14 Children also have a longer at-risk interval for progression to CP, exocrine pancreatic insufficiency, diabetes, impaired growth, and reduced quality of life. 15 As the risk factors accompanying PD in children differ from those in adults, we conducted a systematic review of ERCP for pediatric PD with two aims to : 1) describe the clinical, technical, and safety outcomes that have been reported and 2) determine whether the existing evidence base is capable of supporting conclusions about efficacy.

## Materials and Methods

This systematic review adheres to the guidelines of the updated preferred reporting items for systematic reviews and meta-analyses (PRISMA) 2020 statement. 16 The study protocol was not prospectively registered. Registration was not pursued because the review was planned as a synthesis of a small and predominantly retrospective literature.

### Search Strategy and Study Selection

A systematic search was conducted in PubMed, Ovid, EMBASE, Cochrane, and Scopus up to May 5, 2025, to identify relevant studies assessing the outcomes of ERCP in pediatric patients with PD. Studies were eligible if they reported outcomes of ERCP in patients with PD aged 18 years or younger at the time of the procedure. Studies of mixed adult and pediatric populations were eligible only if outcomes for patients aged 18 years or younger could be extracted separately. Both retrospective and prospective observational studies, in the form of full-text publications and abstracts were included. Review articles, case reports, and case series with fewer than five patients were excluded. We used a combination of medical subject headings terms and free-text keywords related to PD, ERCP, and pediatric populations, combined using Boolean operators across all databases, with no date restrictions. The full search strategy is provided in the Supplement. Two investigators, T.A. and S.K., independently screened citations, resolving discrepancies with a third investigator (S.P.).

### Data Extraction

Data were extracted into a standardized spreadsheet by two independent investigators (T.A. and S.K.), with a third investigator (S.P.) resolving discrepancies. We collected data from each eligible study including authors’ name, publication year, country, type of publication, study design, number of patients, demographics, disease characteristics, follow-up length, ERCP indications, and clinical outcomes. When multiple outcome time points were reported, the latest available follow-up was extracted.

### Outcome Measures

The primary outcome of interest was clinical response after ERCP. When individual studies reported outcomes separately for PD presenting as RAP and as CP, those results were extracted and reported separately. Secondary outcomes were symptom resolution, technical success, and post-ERCP pancreatitis (PEP). For each outcome, the definition used by the source study was used. Each outcome was extracted with the denominator used by the source study, which was the number of patients or the number of procedures depending on the study and the outcome. Therefore, the rates reported by different studies are not always directly comparable.

### Quality Assessment

The Joanna Briggs Institute (JBI) critical appraisal tool, consisting of 10 domains, was used to assess study quality and bias. Responses included “yes,” “no,” “unclear,” and “not applicable.” Studies were classified by risk of bias: low (≥70% “yes” answers), moderate (50–69%), and high (<50%). 17 Two independent investigators (T.A. and S.K.) conducted the assessments, with a third investigator (S.P.) resolving disagreements.

### Data Synthesis

After extraction and interpretation of individual study data, it was determined that quantitative pooling of proportions was not appropriate, as the definition of clinical response and other outcomes differed across studies. The endoscopic interventions differed and were frequently combined within the same cohort, no study included a control group, and outcome ascertainment was incomplete and nonuniform. Study-level results are therefore presented descriptively as counts, proportions, and ranges, and are not pooled. The denominator for each outcome is the number of patients or procedures for whom that outcome was reported by the source study, which is not always the total number of patients in that study.

Ages and follow-up reported by the authors as median and interquartile range (or ranges) were converted into means and standard deviations. The mean age and follow-up time were calculated as the mean of means. 18 A formal evaluation of publication bias was not performed as fewer than 10 studies were available. Assessment of certainty in the body of evidence was performed using the Grading of Recommendations, Assessment, Development, and Evaluation (GRADE) approach for the primary endpoint.

## Results

### Characteristics of Included Studies

Our initial search yielded a total of 731 citations. After deduplication, 313 unique records remained. Title and abstract screening narrowed these to 73 full-text articles for review. Finally, a total of 8 studies with 247 patients were included in this review ( Fig. 1 ). 14 19 20 21 22 23 24 25 All studies were retrospective in design and included pediatric patients with PD undergoing ERCP. Six studies were full-text articles, while two were conference abstracts. In two studies (Agarwal et al and Hsu et al), only the subgroup of patients with PD were included. 19 22 The mean age was 10.7 years, and mean follow-up was 30.1 months. Baseline study characteristics are summarized in Table 1 .

**Fig. 1:**
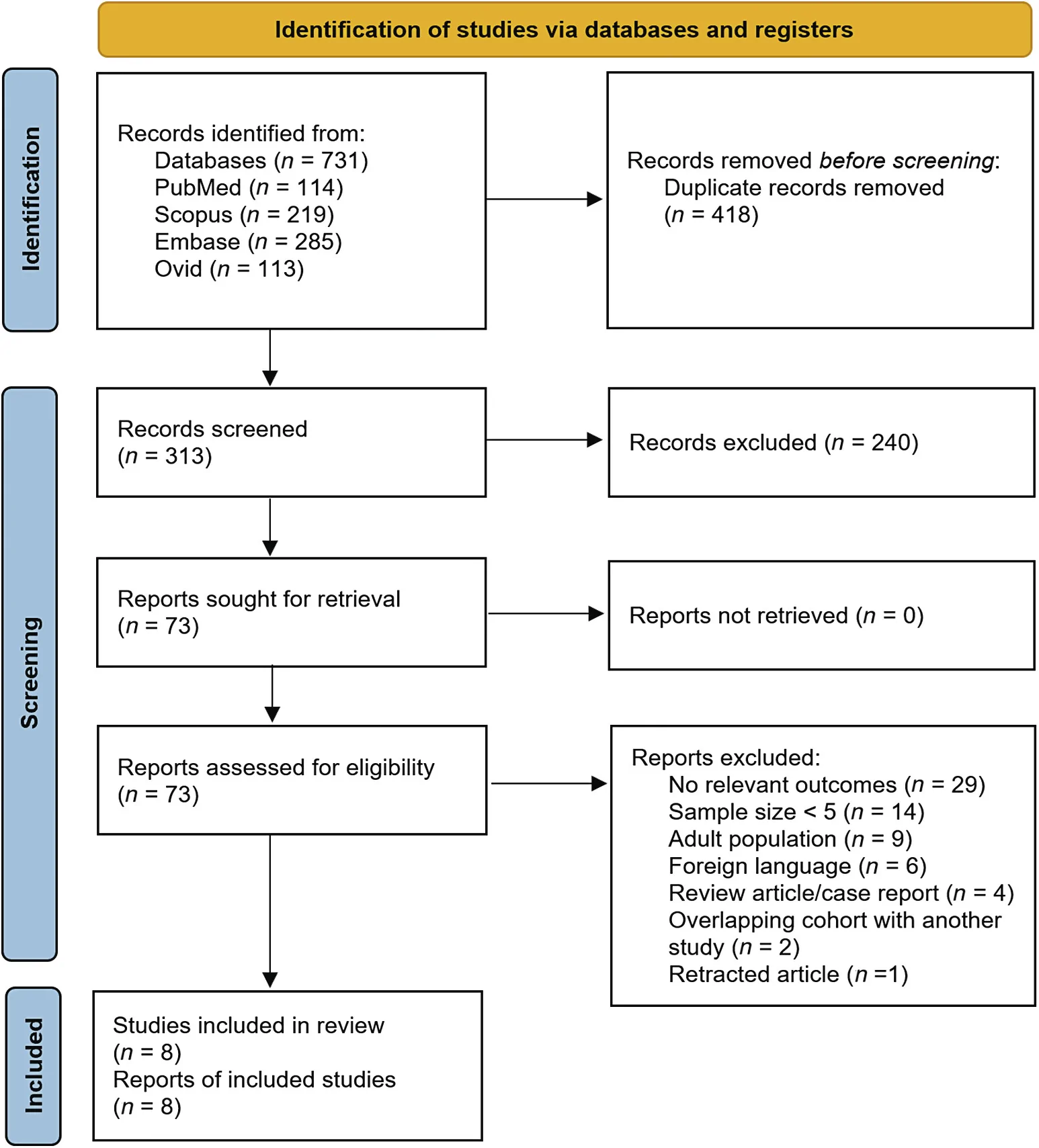
PRISMA flowchart displaying the process of study screening, with the final inclusion of eight eligible studies. 16

**Table 1:** Baseline characteristics of the included studies

Study	Country	Study design	Study period	Procedures, n	Patients, n	Mean age (years)	Males, n (%)	Follow-up duration (months)	RAP sample size	CP sample size	Procedures performed
Agarwal 2013 19	India	Single-Center Retrospective	2010–2011	NR	37*	NR	NR	NR	7	30	MiES, Stent placement
Barakat 2022 14	USA	Single-Center Retrospective	2009–2019	54	54	13.67	13 (24.1)	12	54	NR	MiES
Cui 2018 ^a^ 20	China	Single-Center Retrospective	2011–2017	84	30	NR	NR	38.1	19	11	MiES
Eminler 2023 ^a^ 21	Turkey	Multicenter Retrospective	2014–2023	NR	9	11.7	3 (33.3)	NR	NR	NR	Minor papilla cannulation
Hsu 2000 22	USA	Multicenter Retrospective	1994–1996	NR	6*	10.7	NR	16.4	5	1	MiES, Stent placement, Stone extraction
Lin 2021 23	USA	Single-Center Retrospective	2012–2018	58	27	9.7	14 (51.9)	34.12	6	21	MiES, Dorsal duct cannulation
Pan 2021 24	China	Single-Center Retrospective	2015–2020	114	46	8.6	24 (52.2)	30.73	13	33	MiES, Dorsal duct stent, Dilation
Wen 2019 25	China	Single-Center Retrospective	2008–2017	74	38	9.87	15 (39.5)	49.47	14	24	MiES, Stent placement, Stone extraction

^a^ Conference abstracts.

*Subgroups of original study populations. Pancreas divisum subgroup from Agarwal et al, and pediatric subgroup from Hsu et al

Abbreviations: NR, not reported; MiES, minor papilla endoscopic sphincterotomy

No uniform definition was used for any of the four outcomes, and one study did not state the criteria it applied. The definition used by each study, together with the corresponding result, is reported in Table 2 . Denominators also differ between outcomes within a study, because technical success and PEP were reported per procedure in some studies and per patient in others.

**Table 2:** Outcome definitions and study-level results for each included study

Outcome	Study	Definition applied in the source study	Result, *n* / *N* (%)	Result by presentation, *n* / *N*
Clinical response	Agarwal 2013	Immediate improvement of symptoms with reduction in pain frequency and severity	7/7 (100) ^a^	RAP 7/7
	Barakat 2022	Fewer pancreatitis episodes, less baseline pain, overall milder pancreatitis-associated symptoms	38/47 (80.9)	RAP 38/47 (cohort entirely RAP)
	Cui 2018	Not stated	28/30 (93.3)	RAP 19/19, CP 9/11
	Hsu 2000	Improvement in frequency or severity of pain on the Likert scale	5/6 (83.3)	Not reported separately
	Lin 2021	Reported clinical improvement by parents on questionnaire post-therapy	13/20 (65.0)	RAP 4/5, CP 9/15
	Pan 2021	Reduction of over 50% in the number of acute pancreatitis episodes annually after follow-up relative to pre-treatment frequency	40/46 (87.0)	CP 29/33, RAP 11/13
	Wen 2019	Improvement or resolution of symptoms on the Likert scale, without reintervention or analgesic need after index ERCPs	29/37 (78.4)	RAP 13/14, CP 16/23
Symptom resolution	Barakat 2022	Symptom resolution following ERCP	9/47 (19.1)	–
	Pan 2021	Absence of both abdominal pain and acute pancreatitis after follow-up	24/46 (52.2)	–
	Wen 2019	Resolution of symptoms on the Likert scale without reintervention or analgesic need after index ERCPs	20/37 (54.1)	–
Technical success	Barakat 2022	Successful cannulation and papillotomy	51/54 (94.4)	–
	Cui 2018	Cannulation of minor papilla	82/84 (97.6) ^b^	–
	Eminler 2023	Cannulation of minor papilla	7/9 (77.8)	–
	Lin 2021	Successful deep cannulation of dorsal pancreatic duct	58/58 (100) ^b^	–
	Wen 2019	Deep cannulation of dorsal duct and completion of intended maneuvers	69/74 (93.2) ^b^	–
Post-ERCP pancreatitis	Barakat 2022	New or worsened pain with amylase >3× ULN 24 h post-ERCP and admission or prolonged hospitalization	10/54 (18.5)	–
	Hsu 2000	Not stated	1/6 (16.7)	–
	Lin 2021	New or worsened pain with amylase >3× ULN 24 h post-ERCP and admission or prolonged hospitalization	12/58 (20.7) ^b^	–
	Pan 2021	New or worsened pain with enzymes 3× ULN 24 h post-ERCP	9/114 (7.9) ^b^	–
	Wen 2019	New or worsened pain with amylase >3× ULN 24 h post-ERCP and admission or prolonged hospitalization	10/74 (13.5) ^b^	–

^a^ Agarwal et al reported clinical response only for the subgroup presenting with RAP.

^b^ Reported per procedure. All other results in this table are reported per patient.

Denominators are the number of patients or procedures for whom the outcome was reported by the source study, and are smaller than the total study population where follow-up or outcome ascertainment was incomplete. Studies that did not report a given outcome are omitted from that section of the table.

Abbreviations: RAP, recurrent acute pancreatitis; CP, chronic pancreatitis; ERCP, endoscopic retrograde cholangiopancreatography; ULN, upper limit of normal.

### Primary Outcome: Clinical Response

Six studies, including 186 patients, reported clinical response for their whole PD cohort. 14 20 22 23 24 25 A seventh study, Agarwal et al reported clinical response only for the subgroup presenting with RAP. 19 The definition applied differed in every study and is summarized with the corresponding result in Table 2 . Definitions ranged from a prespecified reduction of over 50% in the annual number of acute pancreatitis episodes 24 to clinical improvement reported by parents on a post-therapy questionnaire, 23 and one study did not state the criteria used. 20 Reported clinical response therefore ranged from 65.0 to 93.3%. Because the definitions and the methods of ascertainment differed across studies, these proportions are not directly comparable and were not pooled.

### Subgroup Analysis

Clinical response was reported by clinical presentation in six studies ( Table 2 ). Response ranged from 80.0 to 100% in RAP 14 19 20 23 24 25 and from 60.0 to 87.9% in CP. 20 23 24 25 Agarwal et al and Barakat et al contribute only to the RAP figures and did not report CP separately. Comparison between presentations is therefore confined to the four studies that reported both, and in three of these, the response was higher in RAP than in CP. 20 23 25 Studies conducted in China reported clinical response ranging from 78.4 to 93.3%, 20 24 25 whereas studies conducted in the USA reported rates ranging from 65.0 to 83.3%. 14 22 23

### Secondary Outcomes

#### Symptom Resolution

Three studies reported symptom resolution over a median follow-up ranging from 12 to 41 months, using three different definitions ( Table 2 ). 14 24 25 The strictest definition required the absence of both abdominal pain and acute pancreatitis at follow-up, so that a child whose pain had settled but who continued to have pancreatitis episodes was not counted as resolved. 24 Reported symptom resolution therefore ranged from 19.1 to 54.1%. The remaining five studies did not report this outcome.

#### Technical Success

Technical success was reported in five studies. 14 20 21 23 25 Two definitions were used. Cui et al, Eminler et al, and Lin et al defined it as successful cannulation alone, of the minor papilla in the first two studies and of the dorsal pancreatic duct in the third, 20 21 23 whereas Barakat et al and Wen et al required cannulation together with completion of the intended maneuver. 14 25 Reported technical success ranged from 77.8 to 100% ( Table 2 ). The denominator was the number of procedures in three of these studies and the number of patients in two, so these results are not additive across studies and no single overall rate can be quoted.

### PEP and Adverse Events

Five studies reported on PEP. 14 22 23 24 25 Barakat et al, Lin et al, and Wen et al applied the same definition, namely new or worsened abdominal pain with amylase greater than three times the upper limit of normal at 24 h after ERCP, together with admission or prolongation of hospitalization. 14 23 25 Pan et al applied the same symptom and enzyme criteria but did not require admission or prolonged hospitalization, 24 and Hsu et al did not state a definition. 22 Reported PEP therefore ranged from 7.9 to 20.7% ( Table 2 ). As with technical success, two studies reported PEP per patient and three reported it per procedure, so these denominators are not additive, and no single overall incidence can be quoted for this literature. Ascertainment was also retrospective in all studies, so the reported rates are likely to be underestimates. Other adverse events were reported by four studies involving 125 patients, with only one case of post-ERCP bleeding reported by Wen et al 14 22 23 25

### Bias Assessment

According to JBI quality assessment criteria, the full-text pediatric PD-specific studies were judged to have low-to-moderate concern within the limitations of retrospective case-series design. Common limitations included retrospective data collection, incomplete follow-up, subjective outcome assessment, and exclusion of patients with failed procedures or unavailable follow-up. In every study, outcomes were assessed by the treating team or by unblinded investigators, and no study used a blinded outcome assessor, which favors the reporting of improvement. Broader pediatric pancreatic ERCP series were judged to have moderate concern because PD-specific outcomes were not always separately reported. The two conference abstracts were judged to have high or unclear concern because of limited methodological detail ( **Table S1** , available in the online version).

### Publication Bias and Certainty of Evidence

Publication bias was not quantitatively assessed due to the limited number of available studies (k = 8). The certainty of evidence for clinical response was rated as very low using GRADE, as all studies were uncontrolled observational series with retrospective designs, variable outcome definitions, and incomplete or subjective outcome assessment. Thus, the reported proportions reflect post-ERCP improvement as recorded in uncontrolled cohorts rather than comparative evidence of ERCP efficacy.

## Discussion and Conclusions

Our systematic review of pediatric patients with PD found reported clinical-response proportions ranging from 65.0 to 93.3% and technical-success proportions ranging from 77.8 to 100%. Symptom-resolution proportions ranged from 19.1 to 54.1% across studies with median follow-up durations of 12 to 41 months. Reported PEP proportions ranged from 7.9 to 20.7%, with denominators varying between patients and procedures. Because all included studies were retrospective and uncontrolled and outcome definitions varied, these figures describe outcomes observed after ERCP rather than causal effects of ERCP.

This review highlights the current limitations of the pediatric PD literature in relation to ERCP. Notably, no study included a control group. Furthermore, among the seven studies that reported clinical response, six applied a definition that differed from that of every other study, and the seventh did not state its criteria at all. The endoscopic interventions also differed, encompassing sphincterotomy, balloon dilation, bougienage, stent placement, and stone extraction, frequently in combination and frequently repeated within the same cohort, so that no single intervention can be linked to any single outcome. The consequence is that the proportions reported in this literature are not directly comparable with one another, and consequently no summary estimate derived from them would have an interpretable referent. Because clinical-response definitions differed, the reported range of 65.0–93.3% should not be interpreted as variation in a common treatment effect. The variety of reported outcomes is nonetheless informative. Across the included studies, reported outcomes included fewer pancreatitis episodes, reduced pain frequency or severity, fewer healthcare encounters, improved general condition, improved nutritional status, and avoidance of or delay in surgery in some patients. 20 22 24 25 Many of these endpoints are important to children and their families and provide meaningful clinical guidance to physicians. These endpoints may be useful when developing future studies; however the current uncontrolled evidence does not clearly establish improvement in outcomes, improves any of them, even in children with a high burden of recurrent pancreatitis.

A plausible mechanism for therapeutic ERCP in pediatric PD is that genetic variants affecting pancreatic secretion or intraductal protease regulation may promote viscous pancreatic secretions, ductal stasis, or ductal plugging. Pan et al specifically noted that genetic abnormalities such as SPINK1, PRSS1, and CFTR may contribute to pancreatic duct blockage through highly viscous pancreatic secretions in children with PD. 24 In a child whose dominant pancreatic drainage occurs through the minor papilla, these secretion-related abnormalities could plausibly amplify the effect of a narrow drainage pathway. This mechanism remains biologically plausible, but unproven, and therefore remains an area of further inquiry. With regard to phenotype, clinical response appeared numerically higher in RAP than in CP, in which ductal distortion, fibrosis, strictures, stones, and potentially centralized pain mechanisms may limit response to drainage-directed therapy. Children with CP may also require repeated interventions, as reported by Wen et al, wherein patients with CP required more ERCPs than those with RAP. 25

The recently published SHARP trial, conducted in adults with RAP and PD, provides the only sham-controlled evidence for minor papillotomy. It did not demonstrate a significant reduction in pancreatitis recurrence occurring more than 30 days after randomization. Notably, both the minor papillotomy and sham groups experienced a similar and substantial reduction in the frequency of pancreatitis episodes after randomization. Because none of the studies included in this review had a control group, the improvement they report may be due to other factors and cannot be attributed to ERCP. Because SHARP enrolled only adults, it cannot directly establish efficacy in children, and is not be fully applicable to this population. Nevertheless, etiologic differences between adult and pediatric disease do not themselves constitute evidence that ERCP benefits pediatric patients. Among the included pediatric PD cohorts, genetic variants were common when testing was performed. Lin et al identified at least one genetic variant in 19 of 26 tested children with PD. 23 Wen et al reported at least one mutation in 44.4% of children with RAP and 68.4% of those with CP, 25 and Pan et al found gene abnormalities in 15 of 26 tested children. 24 These findings support the concept that pediatric PD-associated pancreatitis may reflect an interaction between ductal anatomy and genetic susceptibility rather than PD alone. Key differences between the SHARP adult trial population and the pediatric cohorts included in this review are summarized in Table 3 . 12

**Table 3:** Comparison of population characteristics of SHARP trial and our study population

Domain	SHARP adult RCT	Pediatric cohorts in current review
Study question	Comparative efficacy of minor papillotomy vs. sham ERCP in adults with otherwise unexplained RAP and PD	Reported clinical, technical, and safety outcomes of ERCP in children with PD
Population	Adults with PD and ≥2 AP episodes; other etiologies excluded	Children/adolescents with PD, RAP, and/or CP
Age	Mean age 54 years	Mean age 10.7 years
Pancreatitis burden	Mean lifetime AP episodes 3	Several pediatric cohorts reported frequent annual attacks before ERCP
Etiologic profile	Otherwise unexplained RAP. Patients with high-probability alcohol misuse excluded	Genetic and obstructive risk factors commonly reported
Disease phenotype	Chronic calcific pancreatitis excluded	RAP and CP included
Therapeutic goal	Prevention of recurrent AP after minor papillotomy	Burden reduction: fewer attacks, less pain, improved clinical trajectory, possible delay of surgery
Safety	Acute pancreatitis within 30 days: 14.7% after minor papillotomy vs. 8.2% after sham	Reported PEP incidence 7.9 to 20.7%

Abbreviations: RCT, randomized controlled trial; ERCP, endoscopic retrograde cholangiopancreatography; RAP, recurrent acute pancreatitis; PD, pancreas divisum; CP, chronic pancreatitis; PEP, post-ERCP pancreatitis.

Across the included studies, reported outcomes included fewer pancreatitis episodes, reduced pain frequency or severity, fewer healthcare encounters, improved general condition, improved nutritional status, and avoidance of or delay in surgery in some patients. Regarding safety, the reported PEP range of 7.9–20.7% overlapped the 14.1% pooled incidence reported among high-risk patients in a systematic review of 145 randomized trials, 26 although direct comparison is limited by differences in populations, definitions, ascertainment, and denominators. Because adverse-event ascertainment was retrospective in all included studies, PEP may have been under-ascertained. Furthermore, a meta-analysis of endoscopic therapy for pancreatic divisum in adults showed a notable adverse event rate of 71%. 27 These rates should be interpreted in the context of the procedures performed, since ERCP for PD often requires minor papilla cannulation, dorsal duct manipulation, pancreatic sphincterotomy, dilation, stone extraction, or stent placement, all of which place these procedures in a higher-risk category than routine biliary ERCP. 28 This risk must be considered during patient selection, and further studies are needed to identify patients most likely to have a favorable risk-benefit profile. Data from adult studies support intravenous hydration, pancreatic duct stenting, and rectal nonsteroidal anti-inflammatory drugs to reduce PEP risk, but pediatric-specific evidence remains limited. One retrospective pediatric study reported an association between prophylactic pancreatic duct stenting and PEP, but its observational design does not establish causation. 28 Use of prophylactic strategies was inconsistently reported and generally limited across the included studies ( **Table S2** , available in the online version). 28 29 30

This study has several strengths, including a comprehensive search strategy, PRISMA-based methodology, and synthesis of clinical, technical, and safety outcomes. However, several limitations should be acknowledged. All studies were retrospective, two were conference abstracts with limited methodological detail, and no study included a control group. Therefore, observed improvements after ERCP are difficult to distinguish from natural fluctuations in pediatric recurrent pancreatitis, regression to the mean, spontaneous reduction in attack frequency, or improvement related to other treatments during follow-up. Clinical outcomes were generally assessed without blinding, often by the treating team. Follow-up was incomplete in some cohorts, failed procedures or patients unavailable for follow-up were sometimes excluded, and retrospective adverse-event ascertainment may have missed PEP events. There was also substantial variation in outcome definitions, interventions performed, follow-up duration, and denominators used. Finally, the certainty of the evidence was very low by GRADE.

Progress in this field will therefore require agreement on a core set of outcome definitions for pediatric PD, ideally developed through an established pediatric pancreatitis consortium and applied prospectively. Such a core outcome set should specify what constitutes clinical response and symptom resolution, the time point at which each is assessed, validated pediatric pain and quality-of-life instruments, a uniform definition of technical success, and separate reporting of per-patient and per-procedure adverse events with explicit denominators. Blinded outcome assessment and a control group are equally important because pain and pancreatitis frequency may fluctuate or improve over time without intervention. Genetic testing should also be reported systematically and outcomes should be stratified by variant status. Until these standards are adopted, additional uncontrolled retrospective series are unlikely to resolve whether ERCP benefits children with PD.

In summary, studies of pediatric patients with PD reported high technical-success proportions and frequent study-defined clinical responses after ERCP. However, these observations may not clearly establish whether ERCP is effective because of study design and noncomparable outcome definitions. Given the low certainty of evidence and risk of adverse events, in particular PEP, further prospective studies are needed to define the role of ERCP in this population.

## Data Availability

Template data collection forms, extracted data, analysis datasets, analytic code, and other review materials are available from the corresponding author upon reasonable request.

## Declaration of GenAI Use

Generative AI was used solely to assist with proofreading and grammar. All suggested edits were reviewed and approved by the authors, who take full responsibility for the content of the manuscript.

## Supplementary Material

Ergänzendes Material Supplementary Material
