# Supplementary material for: Endoscopic Retrograde Cholangiopancreatography in Pediatric Pancreas Divisum: Systematic Review
Source: Endosc Int Open. 2026 Oct 27;14:a29776424. doi: 10.1055/a-2977-6424 (PMC13758977; doi:10.1055/a-2977-6424)
Supplement: Supplementary file 1 [file 10-1055-a-2977-6424_29803300.pdf]

Supplementary Material

Tareq Alsaleh, MD, Saurabh Chandan, MD, Shahab R. Khan, MD, Sagar Pathak, MD, Abdul Mohammed, MD, Abdullah Abbasi, MD, Maham Hayat, MD, Natalie Cosgrove, MD, Deepanshu Jain, MD, Kambiz Kadkhodayan, MD, Dennis Yang, MD, Ojasvini C. Chandan, MD, Muhammad K. Hasan, MD, John Goerge, MD, Mustafa A. Arain, MD

Endoscopic Retrograde Cholangiopancreatography in Pediatric Pancreas Divisum: Systematic Review

1. Search strategy

The search strings were provided by an experienced librarian. All searches were conducted on 05/05/2025. No filter for study design, language, or year of publication was used.

PubMed:

("Cholangiopancreatography, Endoscopic Retrograde"[Mesh] OR "endoscopic retrograde cholangiopancreatography") AND ("Pancreas Divisum"[Mesh] OR "pancreas divisum" OR "pancreatic divisum") AND ("Pediatrics"[Mesh] OR "Child"[Mesh] OR pediatric* OR child* OR adolescen* OR teen* OR infant* OR newborn* OR neonat*)

Ovid:

1. exp Cholangiopancreatography, Endoscopic Retrograde/ or "endoscopic retrograde cholangiopancreatography".mp.
2. exp Pancreas Divisum/ or "pancreas divisum".mp. or "pancreatic divisum".mp.
3. exp Pediatrics/ or exp Child/ or pediatric*.mp. or child*.mp. or adolescen*.mp. or teen*.mp. or infant*.mp. or newborn*.mp. or neonat*.mp.
4. 1 and 2 and 3

Embase:

('endoscopic retrograde cholangiopancreatography'/exp OR 'endoscopic retrograde cholangiopancreatography' OR 'ercp') AND ('pancreas divisum'/exp OR 'pancreas divisum' OR 'pancreatic divisum') AND ('pediatrics'/exp OR 'child'/exp OR pediatric* OR child* OR adolescen* OR teen* OR infant* OR newborn* OR neonat* OR 'juvenile'/exp OR 'adolescent'/exp OR 'infant'/exp OR 'newborn'/exp)

Scopus:

(TITLE-ABS-KEY("endoscopic retrograde cholangiopancreatography" OR "ercp")) AND (TITLE-ABS-KEY("pancreas divisum" OR "pancreatic divisum")) AND (TITLE-ABS-KEY(pediatric* OR child* OR adolescen* OR teen* OR infant* OR newborn* OR neonat* OR juvenile OR youth OR children))

Supplementary Tables

Table S1 Risk of bias assessment of the included studies using the Joanna Briggs Institute (JBI) critical appraisal tool.

Study	Q1 Inclusion criteria	Q2 Condition measured reliably	Q3 Valid identification	Q4 Consecutive inclusion	Q5 Complete inclusion	Q6 Demographics	Q7 Clinical/procedural info	Q8 Outcomes reported	Q9 Site/setting	Q10 Statistics	Overall concern
Agarwal 2013	Y	Y	Y	Y	Y	Y	Y	U	Y	Y	Moderate
Barakat 2022	Y	Y	Y	Y	Y	Y	Y	Y	Y	Y	Low
Cui 2018 (a)	Y	U	U	U	U	N	Y	Y	Y	U	High
Eminler 2023 (a)	Y	U	U	U	U	Y	U	Y	Y	U	High
Hsu 2000	Y	Y	Y	U	U	U	Y	Y	Y	Y	Moderate
Lin 2021	Y	Y	Y	Y	Y	Y	Y	Y	Y	Y	Low
Pan 2021	Y	Y	Y	U	N	Y	Y	Y	Y	Y	Low
Wen 2019	Y	Y	Y	Y	N	Y	Y	Y	Y	Y	Low

Table S2 Post-ERCP pancreatitis prophylaxis measures employed in each included study.

Study	IV fluids	PD stent	Rectal NSAIDs
Agarwal 2013	NR	Yes	NR
Barakat 2022	Yes	Yes	Yes
Cui 2018	NR	Yes	NR
Eminler 2023	NR	NR	NR
Hsu 2000	NR	NR	NR
Lin 2021	NR	Yes	NR
Pan 2021	NR	NR	NR
Wen 2019	NR	NR	NR

Abbreviations: NSAIDs, non-steroidal anti-inflammatory drugs; NR, not reported.
